# Diagnostic accuracy of different computer-aided diagnostic systems for malignant and benign thyroid nodules classification in ultrasound images: A systematic review and meta-analysis protocol

**DOI:** 10.1097/MD.0000000000016227

**Published:** 2019-07-19

**Authors:** Ruisheng Liu, Huijuan Li, Fuxiang Liang, Liang Yao, Jieting Liu, Meixuan Li, Liujiao Cao, Bing Song

**Affiliations:** a The First Hospital of Lanzhou University; b The First Clinical Medical College of Lanzhou University; c School of Public Health, Evidence-based Social Science Research Center; d Evidence-based Medicine Center, School of Basic Medical Sciences, Lanzhou University, Lanzhou; e Chinese Medicine Faculty of Hong Kong Baptist University, Kowloon Tong, Hong Kong; f The Second hospital of Lanzhou University, Lanzhou, P.R. China.

**Keywords:** computer-aided diagnosis system, diagnosis, machine learning, meta-analysis, thyroid nodule, ultrasound

## Abstract

**Objective:**

The aim of this study was to determine the diagnostic accuracy of different computer-aided diagnostic (CAD) systems for thyroid nodules classification.

**Methods:**

A systematic search of the literature was conducted from inception until March, 2019 using the PubMed, EMBASE, Web of science, and Cochrane library. Literature selection and data extraction were conducted by 2 independent reviewers. Numerical values for sensitivity and specificity were obtained from false negative (FN), false positive (FP), true negative (TN), and true positive (TP) rates, presented alongside graphical representations with boxes marking the values and horizontal lines showing the confidence intervals (CIs). Summary receiver operating characteristic (SROC) curves were applied to assess the performance of diagnostic tests. Data were processed using Review Manager 5.3 and Stata 15. The methodological quality of included studies was assessed using Quality Assessment of Diagnostic Accuracy Studies (QUADAS-2) tool.

**Trial registration number:**

PROSPERO CRD42019132540

## Abstract 2

Supplemental Digital Content is available in the text

## 1 Introduction

The thyroid nodule, an abnormal growth of thyroid cells that forms a lump within the thyroid gland, is a common clinical problem. It affects 19% to 68% of the healthy population,^[1]^ which about 9% to 15% of thyroid nodules is malignancy.^[2,3]^ Therefore, the early diagnosis of thyroid nodules is clinically important to exclude thyroid cancer and improve the rate of survival. In the United States, approximately 53 990 cases of thyroid cancer were diagnosis in 2018, 13 090 were males and 40 900 were females.^[4]^

American Thyroid Association (ATA) suggests ultrasound (US) should be performed in all patients with known or suspected thyroid nodules.^[5]^ US is the main examination used for both detection and characterization of thyroid nodules^[5–7]^ and facilitate the decision making for fine-needle aspiration (FNA). The following features are usually evaluated by US imaging: thyroid parenchyma (homogeneous or heterogeneous) and gland size; size, location, and sonographic characteristics of any nodule. US imaging characteristics associated with malignant nodules include micro-calcifications, hypoechogenicity, microlobulated or irregular margin, taller-than-wide shape, rich vascularity on color Doppler, and presence of suspicious cervical lymph nodes.^[5,8]^

The main limitation of US is its operator dependence,^[9–11]^ which means that diagnosis results depend on the experience of doctors, level, status, and other factors. Radiologists less experienced are at a greater risk of misdiagnosing a cancer. Moreover, it needs higher clinicians’ qualification for unspecified and indeterminate thyroid nodules while which cannot meet the need in low- and middle-income areas where health resources are scarce. Besides, thyroid nodules continue to be diagnosed with great frequency with largely enhanced diagnostic practices in recent years. A sharp increase in the number of patients has caused a significant increase in the labor intensity among radiologists and a reduction in the average diagnostic time spent on each case, which affects its diagnostic outcome.

In the past 2 decades, many computer-aided diagnostic (CAD) systems have rapidly developed to assist clinical professionals. There are many advantages for the application of CAD system such as improving the accuracy of diagnosis, reducing the time consumption, and decreasing the load of doctors. The CAD system includes 4 phases: image preprocessing, image segmentation, feature extraction, and lesion classification.^[12]^ Nowadays, the accuracy of artificial intelligence in the diagnosis of diabetic retinopathy, liver cancer, ovarian neoplasms, and epilepsy in image recognition achieve great performance.^[13–16]^ Several CAD systems based on pattern recognition methods have been employed for diagnosis of benign and malignant thyroid nodules.^[17–19]^ However, the accuracy of diagnosis varies among different studies using different systems. Some studies indicated the CAD system showed a great diagnosis performance and had a high potential in classifying thyroid nodes.^[20,21]^ But, the study by Gao et al showed the sensitivity of the CAD system in differentiating nodules was similar to than an experienced radiologist while the specificity was lower.^[22]^ The purpose of this study is to evaluate the diagnostic performance of the CAD system in thyroid nodules and to assess its potential role in decision-making alongside radiologists.

## 2 Materials and methods

This review was conducted in accordance with the Preferred Reporting Items for a Systematic Review and Meta-analysis of Diagnostic Test Accuracy Studies (PRISMA-DTA) guidelines,^[23]^ and was registered in the PROSPERO database (International Prospective Register of Systematic Reviews) in May 2019 (registration number CRD 42019132540).

### 2.1 Literature search

A systematic search of the literature was conducted from inception until March, 2019 using the PubMed, EMBASE, Web of science, and Cochrane library. No language, publication date or publication status restrictions were used. The search terms were used the following terminology: ((thyroid nodule or thyroid gland or thyroid) and (computer-assisted or machine learning or deep learning or artificial intelligence or automated) and (diagnosis or diagnos∗ or sensitivity or specificity)). In addition, the references of all eligible studies were manually retrieved to ensure the comprehensiveness of the search. The detailed search strategies for PubMed were presented in Supplemental Table S1.

### 2.2 Inclusion criteria

**Figure 1:**
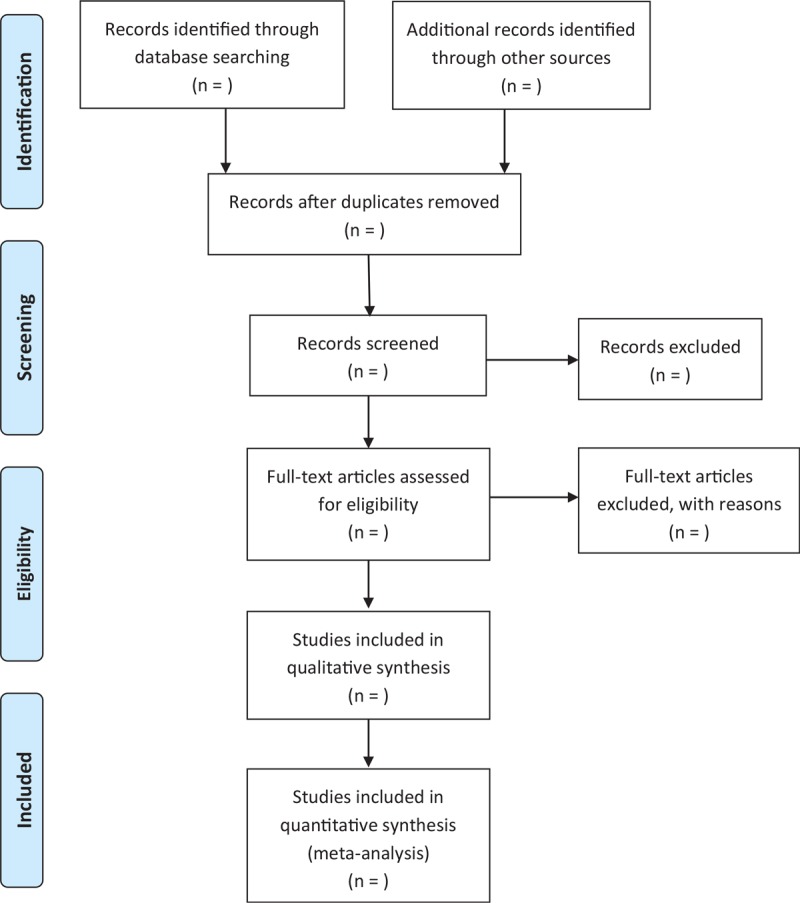
Flow diagram of study selection process.

Studies were eligible for inclusion if they evaluated the diagnostic accuracy of a CAD system—either a complete algorithm or deep learning-features—to classify benign and malignant thyroid nodules using US images. Patients with thyroid nodules with decisive diagnosis were recruited. The primary outcomes included the performance of the computer-aided diagnosis systems for diagnosis of malignant thyroid nodules, included accuracy, sensitivity, specificity, positive predictive value, and negative predictive value. We excluded duplicates, reviews, comments, editorials, conference abstracts, and unpublished articles. The selection process will be presented in a PRISMA flow diagram (Fig. 1).

### 2.3 Literature selection and data extraction

The retrieved records were imported into the EndNote X7 software and the duplicate publications were excluded. Two reviewers (LHJ and LMX) independently read the titles and abstracts of all identified records to exclude those that were clearly not relevant. Then the full texts of the articles retained were reviewed to further determine their suitability. Differences opinions were resolved by consensus.

The data were extracted by 2 reviewers (LHJ and LMX) independently using a pre-defined form. The following characteristics of included studies were collected: the first author, publication year, country, number of included mages/patients, training dataset, validation dataset, CAD system, accuracy, sensitivity, specificity, and main conclusion. Any discrepancies were resolved by consensus.

### 2.4 Risk of bias assessment

The methodological quality of included studies was assessed using quality assessment of diagnostic accuracy studies (QUADAS-2) tool.^[24,25]^ Four key domains were evaluated, including patient selection, index test, reference standard and flow and timing. Each domain was assessed in terms of risk of bias and in the first 3 in terms of concerns regarding applicability. The risk of bias was examined by 2 reviewers concurrently, and discrepancies were resolved by consensus. The detailed domains for risk of bias assessment were presented in Supplemental Table S2.

### 2.5 Statistical analysis

The paired forest plot analysis was generated by using mock data. Numerical values for sensitivity and specificity were obtained from false negative (FN), false positive (FP), true negative (TN), and true positive (TP). They were presented alongside graphical representations which the boxes marked the values and the horizontal lines showed the confidence intervals (CIs). The summary receiver operating characteristic (SROC) curve represented the performance of a diagnostic test. Subgroup analysis was performed for different computer-aided diagnosis systems. Data were analyzed by using Review Manager 5.3 and Stata 15. However, if quantitative synthesis was not appropriate, meta-analysis could not be conducted. Instead, evidence was summarised in narrative form.

## 3 Discussion

US examination is a safety, convenience, and low cost for the diagnosis of thyroid nodules. The CAD systems have been developed to assist the radiologist in the image interpretation, speed up the diagnostic process and reduce interobserver variability.^[26]^ Previous studies have reported the usefulness of CAD for thyroid nodule. This systematic review focus on the diagnostic performance of different CAD system that is currently applied in thyroid nodule diagnosis and assessment its potential role in decision-making alongside radiologists.

### 3.1 Strengths and limitations

This study has several strengths. First, our systematic review will be the first to evaluate the diagnostic performance of the CAD system in thyroid nodules. Second, we performed comprehensive search to identify studies on the diagnostic accuracy of a CAD system for thyroid nodules using US images. Third, we assessed the quality of included studies by 2 reviewers independently using QUADAS-2 and presented the evidence quality of studies. However, there are still some potential limitations. Only English language articles were included, which might not fully applicable for studies in other language. Moreover, only CAD systems based on the US imaging were included in consideration of consistency among different studies. The other CAD systems using the cytological images were excluded.

## Author contributions

**Conceptualization:** Fuxiang Liang, Liang Yao, Bing Song.

**Data curation:** Fuxiang Liang, Meixuan Li, Liujiao Cao.

**Formal analysis:** Liang Yao.

**Investigation:** Jieting Liu, Meixuan Li.

**Methodology:** Huijuan Li, Liang Yao, Liujiao Cao.

**Resources:** Jieting Liu.

**Software:** Liujiao Cao.

**Visualization:** Bing Song.

**Writing – original draft:** Ruisheng Liu, Huijuan Li.

**Writing – review & editing:** Ruisheng Liu, Bing Song.

## Supplementary Material

Supplemental Digital Content
