# Supplementary material for: Diagnostic accuracy of different computer-aided diagnostic systems for malignant and benign thyroid nodules classification in ultrasound images: A systematic review and meta-analysis protocol
Source: Medicine (Baltimore). 2019 Jul 19;98(29):e16227. doi: 10.1097/MD.0000000000016227 (PMC6709132; doi:10.1097/MD.0000000000016227)
Supplement: Supplemental Digital Content [file medi-98-e16227-s001.doc]

Table S1. Search strategies in PubMed.

| **Number** | **Search terms** |
| --- | --- |
| #1 | "Thyroid Nodule"[Mesh] OR "Thyroid Gland"[Mesh] OR Thyroid[Title/Abstract] OR Thyroid Gland[Title/Abstract] OR Thyroid Nodule[Title/Abstract] OR thyroid nodules[Title/Abstract] OR thyroid Cancer[Title/Abstract] OR thyroid tumour[Title/Abstract] OR thyroid Neoplasms[Title/Abstract] OR Thyroid Carcinoma[Title/Abstract] |
| #2 | Artificial Intelligence[Title/Abstract] OR Deep learning[Title/Abstract] OR Computer-Assisted[Title/Abstract] OR Machine learning[Title/Abstract] OR neural network[Title/Abstract] OR neural network*[Title/Abstract] OR Artificial intelligence [Title/Abstract] OR Computational Intelligence[Title/Abstract] OR Machine Intelligence[Title/Abstract] OR Computer Reasoning[Title/Abstract] OR Automated[Title/Abstract] |
| #3 | "Diagnosis"[Mesh] OR Diagnosis[Title/Abstract] OR Diagnos*[Title/Abstract] OR Sensitivity[Title/Abstract] OR Specificity[Title/Abstract] OR Accuracy[Title/Abstract] OR Positive likelihood[Title/Abstract] OR negative likelihood[Title/Abstract] OR ROC[Title/Abstract] |
| #4 | #3 AND #2 AND #1 |

Table S2 QUADAS-2.

| **Domain** | **Questions** |
| --- | --- |
| Domain 1. Patient Selection | A. Risk of Bias  Patient Sampling  Was a consecutive or random sample of patients enrolled?  Was a case-control design avoided?  Did the study avoid inappropriate exclusions?  Could the selection of patients have introduced bias?  B. Concerns regarding applicability  Patient characteristics and setting  Are there concerns that the included patients and setting do not match the review question? |
| Domain 2. Index Test(s) | A. Risk of Bias  Were the index test results interpreted without knowledge of the results of the reference standard?  If a threshold was used, was it pre-specified?  Could the conduct or interpretation of the index test have introduced bias?  B. Concerns regarding applicability  Are there concerns that the index test, its conduct, or interpretation differ from the review question? |
| Domain 3. Reference Standard | A. Risk of Bias  Is the reference standards likely to correctly classify the target condition?  Were the reference standard results interpreted without knowledge of the results of the index tests?  Could the reference standard, its conduct, or its interpretation have introduced bias?  B. Concerns regarding applicability  Are there concerns that the target condition as defined by the reference standard does not match the question? |
| Domain 4. Flow and Timing | Was there an sppropriate interval between index test and reference standard?  Did all patients receive the same reference standard?  Were all patients included in the analysis?  Could the patient flow have introduced bias? |
